# Mechanobiology of Platelets: Techniques to Study the Role of Fluid Flow and Platelet Retraction Forces at the Micro- and Nano-Scale

**DOI:** 10.3390/ijms12129009

**Published:** 2011-12-07

**Authors:** Shirin Feghhi, Nathan J. Sniadecki

**Affiliations:** 1 Department of Mechanical Engineering, University of Washington, Stevens Way, Box 352600, Seattle, WA 98195, USA; E-Mail: shfeghhi@uw.edu; 2 Department of Bioengineering, University of Washington, 3720 15th Ave NE, Seattle, WA 98105, USA

**Keywords:** platelet aggregation, platelet forces, shear flow, BioMEMS, microfluidics

## Abstract

Coagulation involves a complex set of events that are important in maintaining hemostasis. Biochemical interactions are classically known to regulate the hemostatic process, but recent evidence has revealed that mechanical interactions between platelets and their surroundings can also play a substantial role. Investigations into platelet mechanobiology have been challenging however, due to the small dimensions of platelets and their glycoprotein receptors. Platelet researchers have recently turned to microfabricated devices to control these physical, nanometer-scale interactions with a higher degree of precision. These approaches have enabled exciting, new insights into the molecular and biomechanical factors that affect platelets in clot formation. In this review, we highlight the new tools used to understand platelet mechanobiology and the roles of adhesion, shear flow, and retraction forces in clot formation.

## 1. Introduction

**Figure 1:**
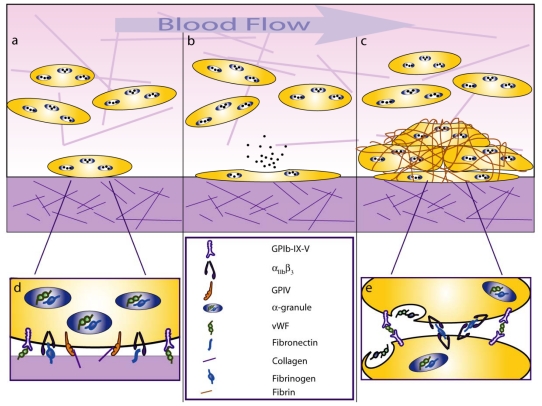
Platelet Adhesion and Aggregation: (**a**) Platelets adhere to the vessel wall when exposed to matrix proteins. (**b**) Adhered platelets undergo shape change and release soluble adhesive proteins from their α-granules. (**c**) A hemostatic plug is formed when platelets adhere to fibrin and each other. Specific receptor-ligand bonds mediate (**d**) platelet adhesion and (**e**) platelet aggregation.

Platelets are one of the smallest cells in the human body, having discoid shapes with 2–4 μm diameters, but they play a large role in preventing blood loss when damage has occurred in a vessel [1,2]. Platelets initiate hemostasis by using their glycoprotein receptors to form attachments to the damaged tissue, which arrests them from circulating in the blood (Figure 1a) [3,4]. Once attached, platelet release a variety of agonists and soluble adhesive proteins from within their granules to activate and recruit more platelets to the wound site (Figure 1b) [5]. Platelets can also act as biomechanical elements for the growing clot structure by using their glycoprotein receptors to form bridges between other platelets and the surrounding protein meshwork that forms the hemostatic plug (Figure 1c). They further reinforce the integrity of the plug by using their cytoskeletal filaments to undergo shape change [6,7], forming protrusions that enable more physical connections with other platelets within the clot, while also using their actin-myosin interactions to pull the clot into a more compact structure that stabilizes it against the vessel wall [8].

Glycoprotein receptors in platelets bind to ligands sites found within the extracellular matrix (ECM) of the vessel wall and soluble adhesive proteins that platelets release [9]. ECM proteins of the vessel wall consist mainly of collagen and laminin, but soluble adhesive proteins like von Willebrand Factor (vWF), fibrinogen, and fibronectin can also deposit onto the wound site to enhance platelet adhesion (Figure 1d) [10–13]. The initial arrest of a platelet from the blood flow involves the glycoprotein receptor GPIb-IX-V, but subsequent engagement of GPIV to collagen can activate integrins α_2_β_1_ and α_IIb_β_3_, which further assist in the adhesion process. P-selectin receptors on the surface of activated endothelial cells can mediate platelet adhesion through interactions with P-selectin glycoprotein ligand 1 (PSGL1) on a platelet’s membrane after degranulation [14,15]. Moreover, platelet GPIbα receptors can also interact with P-selectin to aid in homing platelets to the site of injury [16]. Receptor-ligand interactions are nano-scale and a single platelet can have a multitude of different receptor-ligand interactions during clot formation (Figure 1e). Understanding these small and complex interactions requires approaches that can specifically control the ligand presentation on a surface and have sufficient measurement sensitivity to interrogate their biophysical properties.

Physical forces also play a critical role in hemostasis by regulating the mechanobiology of platelets. When a platelet adheres to a wound site, adhesive forces keep the platelet attached and prevent it from being dislodged by the blood stream. Receptors GPIb-IX-V and α_IIb_β_3_ are known to have a large role in platelet mechanobiology because they regulate the initial tethering to the vessel wall and the activation of platelet shape change and force generation [17]. Upon activation, G-actin monomers in platelets polymerize into F-actin filaments, allowing platelets to undergo shape change. Platelet activation also leads to phosphorylation of non-muscle myosin, which can in turn, engage with actin and form contractile filaments. The contractile forces produced by platelets are in the range of piconewtons for a single actin-myosin complex to nanonewtons for single platelets, but are vastly important in stabilizing a clot by compacting its structure [18] and in strengthening platelet adhesions through integrin-related mechanotransduction [19]. Another type of force that is important in hemostasis is shear forces applied to platelets due to flow of blood. Shear forces can cause platelets to detach, but are also known to have a major role in the steps from platelet adhesion to aggregation.

A multitude of engineered devices have been developed to look at adhesive, contractile, and shear forces and the role of agonist and receptor-ligand bindings on the clot formation process [5,20–25]. Among the technological advances for studying platelets, micro- and nano-scale tools have been used recently to understand platelet biology and thrombus formation dynamics [24,26,27]. The advantage of these tools is that platelets and their adhesion receptors are micro- and nano-scale is size, so devices that are in the same size range as platelets can be used as programmable materials, in which the physical and adhesive interactions between platelets and their surroundings can be controlled and measured. In this review, we will highlight the tools used to examine clot formation with an emphasis on the tools used to study the role of hemodynamic shear and platelet forces.

## 2. Platelet-Shear Flow Interactions

Early studies on platelet adhesion and aggregation were conducted in the absence of shear flow [28–30]. Soluble factors were assumed to be the main mechanisms driving hemostasis and that shear flow had a minor effect. These studies on platelets under static conditions examined the effect of different ECM proteins and biomaterials on platelet adhesion, shape change, and spreading, and the role of different agonists and inhibitors on platelet aggregation [30,31]. These studies were helpful in gaining a better understanding of the process, but shear forces were later recognized as having a more fundamental role in platelet adhesion and aggregation process, rather than simply transporting platelets to the vessel wall through collisions with red blood cells [9,32,33]. Moreover, many of the findings from the static assays were found to be different under shear flow conditions, including biocompatibility of some of the materials used for stents, heart-valves and grafts [34].

**Figure 2:**
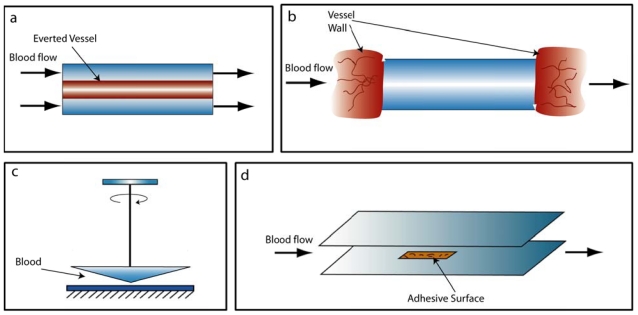
Conventional Flow Devices: (**a**) Annular flow chamber, (**b**) Tubular flow chamber, (**c**) Cone and plate flow device, and (**d**) Parallel plate flow chamber.

Shear forces are produced by the fluid layers of blood passing by each other at different velocities and therefore applying a shearing force on particles in the flow. Shear rate is used to describe this gradient of velocities within a flow. Typically, shear rates are low in large vessels (<600 s^−1^) and are high in the vessels with smaller diameters (up to 5000 s^−1^). Depending on the shear rate, different platelet receptors can dominate the adhesive interaction with the vessel wall or other platelets, e.g.. α_IIb_β_3_ strongly affects adhesion under low shear rate (<1000 s^−1^), while GPIb-IX-V dominates under high shear rates (>10,000 s^−1^) [35]. Therefore, to better understand platelet adhesion and aggregation, a number of perfusion chambers have been developed for *ex vivo* and *in vitro* studies (Figure 2) [36]. These chambers can be configured to mimic the flow velocities and rheological properties of blood flow in an experimental setting, enabling one to study a range of shear forces and shear rates that platelets experience *in vivo.* Additionally, surface modifications such as different ECM coatings are possible and easy access to the chamber is provided, which can be advantageous for introducing different chemicals such as agonists and inhibitors into the flow.

### 2.1. Conventional Devices

*Annular flow chambers* were an early design used to study platelet adhesion as well as thrombus formation under *ex vivo* flow conditions (Figure 2a,b) [37–39]. A segment of human or rabbit vessel was turned inside-out and fixed to a rod in the middle of a larger cylinder. Whole blood could be pumped through the cylinder in order to study platelet and fibrin deposition. The benefit of using the annular chamber was that it allowed for a large range of control over the shear rate, which helped to identify the importance of vWF-GPIb and fibrinogen-α_IIb_β_3_ interactions under higher shear rates [40–43]. The downside however was that since the adhesive wall was a vessel segment, platelets were exposed to the native ECM, which had an uncharacterized composition of ligands with which the platelets could interact, making it difficult to study specific receptor-ligand interactions.

*Tubular flow chambers* allowed for better control over the adhesive interactions, but it did not have the same control over the shear rate as a result of the *ex vivo* nature of the assay. The typical setting of these assays consists of a tube coated with the targeted ECM or anticoagulant and surgically inserted between an artery and vein to form a shunt. This approach allowed for studies on platelet binding to surfaces with well-defined ECMs [44]. In addition, the tubular devices were helpful in studying drug effects on platelets due to the usage of blood in the absence of anticoagulants. Different synthetic graft and stent materials could be examined using these devices because of the particular bio-compatibility issues that they can introduce when exposed to the blood and the potential for clot formation possibly leading to restenosis, embolism, or other secondary defects [45–47].

*Cone and plate flow* devices are another type of devices used to expose platelets to uniform and well-defined shear rates (Figure 2c) [38,48]. To run the assay, a sample of blood or platelet-rich plasma is placed between a rotating cone and a stationary well, which exposes the sample to the shear rate that is determined by the rotation speed and the angle of the cone [49,50]. Cone and plate flow devices have been used to investigate platelet adhesion and aggregation on different ECMs [51–53]. More recently, these devices have been used to produce more complicated flow regimes, such as pulsatile shear stresses that mimic stenosed regions or recirculation zones [54]. Moreover, these devices has been combined with an upright epi-fluorescence microscope to allow for real-time studies of the thrombogenecy of biomaterials [55]. One shortcoming of this assay is that the open surface at the rotating cone can lead to evaporation of the sample.

*Parallel plate flow chamber* is used most frequently among the types of flow perfusion systems (Figure 2d) [56,57]. The early design was a channel with a cover-glass holder at the bottom where different ECMs could be introduced to the system using a cover-glass that was pre-treated with adhesive proteins. After exposure to flowing platelets or whole blood, the cover-glass can be removed, fixed, stained, and analyzed for the platelet adhesion and thrombus formation [58]. Later with the usage of new imaging techniques, channels were made of clear and thinner materials to allow for a microscope objected to be close enough for live-microscopy studies of platelet adhesion and thrombus formation [11,22]. An advantage of parallel plate flow chambers over the other devices is that they can be modified to mimic different *in vivo* conditions; among these, pulsatile flow [59] and disturbed flow [60] can be generated with a slight modifications to pumping system or flow chamber design. Porous membranes can also be used instead of cover-glass to allow for controlled introduction of chemicals to the system through the membrane [61]. These membranes help to mimic the release of different chemicals from endothelial cells such as agonists and anticoagulants. One limitation with parallel plate flow chambers is the use of anti-coagulated blood in these studies; to overcome this limitation, these chambers have also been used *ex vivo* by surgically inserting them into the subject’s body in order to study the thrombogenic characteristics of grafts and stents [62]. Parallel plate flow chambers have been very successful in determining many of the unknowns in platelet adhesion and aggregate formation, including the effect of shear on platelet-ECM binding mechanisms as well as clot formation and stability [7,10,11,21,22,63,64].

### 2.2. Microfluidic Devices

Miniaturized versions of parallel plate flow chambers have been developed to improve the micro-and nano-scale capabilities for studying platelets under shear flow [65,66]. These microfluidic devices are typically made of a silicone rubber (polydimethylsiloxane, PDMS), which can be cast against a silicon master to replicate its features [67]. This process is referred to as soft lithography and allows for high precision in replicating micro- and nano-scale features that have been built onto a master silicon wafer using processing tools for semiconductor fabrication [68]. To build the microfluidic devices, multiple layers of PDMS are bonded together, where each layer contains a part of the design of channels and posts [69]. This method is quite versatile and a variety of unique and creative chamber configurations have been achieved.

**Figure 3:**
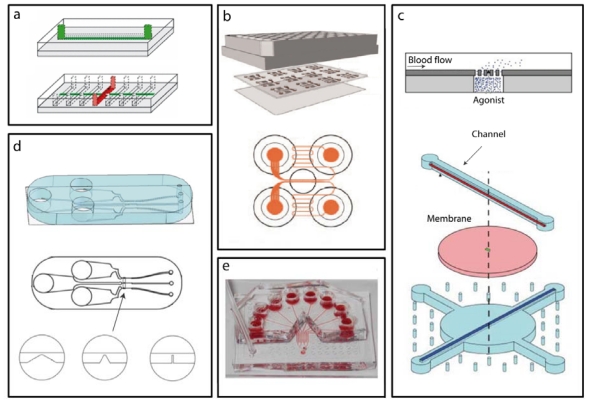
Microfluidic Devices: (**a**) A microfluidic device with seven parallel microchannels (red) to run simultaneous experiments under different shear rates. A horizontal channel (green) is used to flow ECM proteins in solution and deposit the protein as adhesive patches on the array of horizontal channels used for blood flow. Adapted with permissions from [77]. (**b**) A compact array of microfluidic devices allows the operator to run multiple experiments simultaneously. Adapted with permissions from [82]. (**c**) A microfluidic device used for the controlled release of agonist through a membrane structure at the bottom of the flow channel. Adapted with permissions from [73]. (**d**) A microfluidic device with three different types of obstructive geometries in the main channel is used to examine the effect of gradients in the strain rate. Adapted with permissions from [75]. (**e**) A microfluidic device used to expose the same sample of blood to different inhibitors. Adapted with permissions from [83].

Microfluidic devices have been used in biological studies because they have the capability to mimic the *in vivo* conditions closely. They have been used effectively in vascular research studies, e.g., shear flow mechanotransduction, cell-cell interactions, cell migration, and targeted drug delivery [70,71]. In particular, they have been used to confirm the observations made using conventional flow devices, but at the same time, they provide powerful insights into the interactions between platelet receptors and matrix ligands under different shear rates [10,12,72]. Additionally, they have been used to study clot formation and the role of different agonists [73], anticoagulants [74], strain micro-circulation zones [75] (Figure 3).

These devices have been able to successfully resolve one of the major short comings of conventional flow chambers, which is the high volume consumption of sample blood and reagents. Human subjects provide adequate volumes of sample blood to run assays using parallel plate flow chambers, but in some cases, a sample volume of blood is limited, e.g., sickle cell anemia. Blood from mice, which is advantageous for studying genetic modification, has major limitations in sample volume since an average-sized adult mouse can yield less than 1 mL of blood volume [76]. In comparison, microfluidic devices require lower volumes of sample blood, usually less than 50 μL [73,77–79]. The lower volumes possible with microfluidics have expanded the studies on platelets from knock-out mice, which in turn have provided more insights on the role of platelet receptors in adhesion and aggregation [79,80].

Microfluidic devices have also been helpful in running assays at different shear rates [81]. A variety of environments can be introduced under these shear rates, using patterning techniques to create wound-like environments with well-defined areas of adhesive protein (Figure 3a) [77]. They have enabled studies on aggregate formation to be run with multiple experiments simultaneously (Figure 3b) [74,82] or with controlled concentration of agonists (Figure 3c) [73]. Of particular noteworthiness, a novel microfluidic device has been used to investigate aggregate formation after exposure to micro-gradients in the shear rate (Figure 3d) [24,75]. In these studies, the walls of the microchannel were built with a section that narrowed and then expanded in width as the flow passed over an obstruction in the channel. This configuration was used to generate a gradient in the shear rate that was similar to what platelets experienced as they pass through a stenosis *in vivo*. Changes in the shear rate were able to initiate platelet adhesion and aggregation, even when the platelet response to conventional agonists was blocked. The implications of these studies suggest that biomechanical factors can activate platelets independent of biochemical factors.

Finally, microfluidic devices have been used to run parallel assays under different conditions [74,77,82]. Due to the fact that they work with very low blood volumes they can be used to run parallel varying a parameter such as shear rate [77,84], chemicals including inhibitors (Figure 3e) [83], and matrix coatings [82]. The advantage of parallel microchannels is that they significantly reduce the overall assay time since all experiments can be run simultaneously. They also help to reduce the sample-to-sample variability and effects of other randomly introduced factors such as operator handling because the experiments are runs at the same time in the same device. Therefore, microfluidic assays afford a higher degree of reliability in assessing platelet functions or aggregate formation assays when varying environment conditions.

## 3. Platelet Clot Retraction

**Figure 4:**
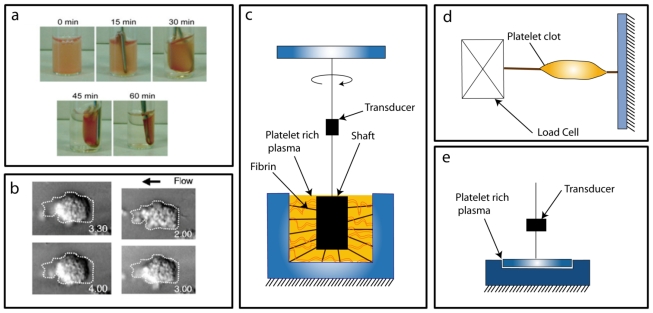
Conventional Platelet Force Assays: (**a**) Clot retraction assay. Adapted with permissions from [88]. (**b**) *In-vitro* observations of thrombus consolidation. Adapted with permissions from [25]. (**c**) Thromboelastography. (**d**) Clot strip assay. (**e**) Clot retractometry.

Clot retraction is the final step in platelet aggregate formation. Platelets pull on fibrin strands to consolidate the size of the hemostatic plug to allow the flow of blood to recommence. Consolidation of the plug reduces the fluid drag acting on it [85–87], but also leads to tighter association between platelets and fibrin strands that prevents fibrinolysis [18]. Moreover, actin-myosin force generation in platelets helps stabilize their receptor-ligand adhesions through integrin-related mechanotransduction [19]. To examine clot retraction, a number of conventional methods have been developed that are briefly discussed here (Figure 4).

### 3.1. Conventional Force Assays

*Clot refraction assay* is a common method to assess consolidation by monitoring the volume change in a thrombus *in vitro.* This method is simple to implement because platelet-rich plasma is placed in a vial with a metal rod and agonists are added to initiate the thrombus formation (Figure 4a). As the thrombus consolidates around the rod, plasma is extruded from its volume. The degree of retraction is determined by removing the thrombus from the vial and measuring the remaining plasma volume. This method has provided useful insights into the important role of α_IIb_β_3_ and its integrin-related kinases in clot retraction [88–90]. Recently, this method has been adapted for microscopic studies where the volume change of a micro-clot is monitored by tracking the movement its platelets as they pull closer together (Figure 4b) [25]. However, the general shortcoming of the clot retraction method is that it does not allow for the direct measurement of platelet forces, but merely the volume change association with clot retraction.

*Thromboelasrography* examines clot retraction by measuring the viscoelastic characteristics of blood during clot formation under low shear (Figure 4c) [91–94]. The system consists of a stationary cup that holds the sample and is rotated back and forth by a small angle. A pin is suspended in the blood sample and is normally stationary, but when fibrin strands and platelet adhesions formed, the torque from the rotating cup is transferred to the pin, which then rotates. The strength of these fibrin strands and platelet forces affect the motion of the pin. Its rotation is measured by a transducer and reported as the output of the system. Key information obtained by thromboelastography consists of the initiation of coagulation, propagation kinetics, fibrin-platelet interaction, clot firmness, and fibrinolysis [93]. The main shortcomings of this method, however, are its sensitivity to mechanical vibrations and long assay time in the absence of chemical agonists. To address these shortcomings a variation of this method called rotating thromboelastography (ROTEM) has been designed which transmits the pin signal through an optical detector and the movement is initiated by the pin [95]. This technique is considered a whole blood coagulation assay and is used to predict surgical bleeding and aid to determine blood product usage for a patient clinically [91].

*Platelet clot strips* was the first technique to directly examine contractile forces within the clots (Figure 4d). Clot strips are formed by pouring platelet-rich plasma into a cylindrical tube and activating them with thrombin or heating up the clots [96–98]. After removing the clots strip from the tube, one end is tied to a rigid support and the other is tied to a load cell which monitors the retraction force in the sample. This technique has been used to study the effect of different agonists on the tension generated in a clot [96], receptors involved in fibrin-platelet binding [99], and alignment of fibrin in the clot contraction [97]. The positive aspect of this method is that since the assays are done in a fluid bath, it is possible to change the conditions of the experiment by changing the bath solution. Its shortcoming, however, is that the clot needs to be directly manipulated when it is mounted onto the load cell, resulting in mechanical disruption of the fibrin-platelet adhesions which can compromise the testing results.

*Clot retractometry* examines platelet clots by directly forming them between a cup and plate which is coupled to a strain gauge transducer with a voltage output (Figure 4e) [100,101]. The plate is in contact with the blood sample and during clotting, fibrin strands as well as platelet aggregates connect the cup and plate. Once clots are formed, platelets forces pull on the fibrin strands, which in turn pull down on the plate. This movement is then transferred to a load cells that reports the output of the system. The advantage that this method over others is that it allows for measurement of the force at the onset of clot formation and does not require additional physical handling of the growing clot. Clot retractometry has been used extensively to investigate the clinical relevance of platelet forces in cardiovascular disease and medical treatments [101–110].

### 3.2. Micro/Nano Force Assays

Conventional force measurement techniques discussed here have been successful in demonstrating the importance of platelet forces in hemostasis. There is, however, an inherent size and resolution limitation within these methods. Platelet cytoskeleton-fibrin interactions are nanoscale and complex with glycoprotein receptors playing different roles in adhesion, aggregation, and clot retraction. The macro-scale dimensions of the conventional techniques do not allow for microscopic imaging, which can help reveal the dynamic features of thrombus formation. Moreover, the fibrin meshwork has strain-stiffening behavior under load, which confounds the direct measurement of platelet forces. Therefore, researchers have begun to explore tools that come from micro- and nano-scale technologies in order to gain more insight into the mechanobiology of platelets.

**Figure 5:**
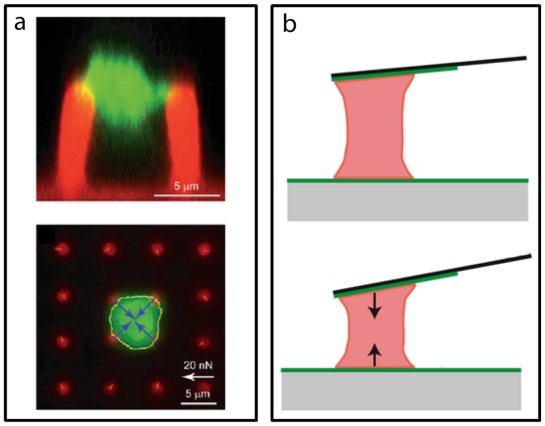
Micro/Nano Platelet Force Assays: (**a**) Micropost arrays. Top: side-view of a platelet clot (green: actin) on two microposts (red: DiI). Bottom: top-view of a platelet clot on four microposts within an array. Platelets produced contraction forces that bend the microposts (blue arrows). Adapted with permissions from [26], (**b**) Atomic force microscopy (AFM). Top: AFM tip is in the original position because the platelet attached to it has not contracted. Bottom: AFM tip is bent by the contraction force of the platelet. Adapted with permissions from [27].

*Micropost arrays* are a novel microscale force sensor that have been used previously to measure cellular traction forces [111,112]. This sensor is an array of micro-size, flexible, vertical posts that bend in proportion to the forces that cells apply at tips of the posts. The posts are made from PDMS using soft lithography, similar to the fabrication of microfluidic devices. These arrays have been used to study cell migration [112], cell spreading [111], and traction forces [111,113–118]. Forces within monolayers [112,119–122] as well as tissue constructs [123], and cell-cell forces [124,125] have also been examined using this tool. At the same time a variety of cell studies have been done using different types of cells, such as fibroblasts [111,116,117,126,127], smooth muscle cells [111,128], cardiomyocytes [129,130], epithelial cells [115,126], endothelial cells [128], and stem cells [114,131]. Micropost arrays are considered a novel tool for cell mechanic studies because they can be used to map the traction forces of cells spread over multiple posts. Additionally, live studies are possible with microposts to investigate the dynamics of cytoskeletal force development. The unique property of this sensor is that it can be bio-functionalized with different adhesive proteins to study specific receptor-ligand interactions. Micropost arrays have been recently used to investigate platelet forces in more detail (Figure 5a). The arrays have been used to examine the effect of concentration of thrombin on contractile forces of platelet aggregates as well as the adhesive interactions with fibronectin and fibrinogen [26]. Comparisons also have been done between quasi-static and live imaging of aggregate formation to show its spatio-temporal capability in assessing platelet functionality and thrombus formation.

*Atomic force microscopy* (*AFM*) is another technique that is typically used for nano-scale characterization of materials, but it has been adapted to study the mechanical properties of cells and single molecules [132]. AFM uses a flexible tip that acts like a cantilever to measure forces in the vertical direction. The deflection of the tip is measured from a laser beam that is reflected off the back of the tip and towards an array of photodiode detectors. AFMs have been used to investigate cell mechanics and cytoskeletal elasticity [133–140] as well as cell-cell forces [141–143], receptor-ligand dynamic interactions [141,144], and cytoskeletal proteins [145,146]. Recently, AFM has been used to study the nature of the ligand-receptor bond between vWF and GPIbα in platelets. Platelet adhesion to the vessel wall likely involves catch-bonds between vWF and GPIbα because high shear stress leads to greater binding [147]. This observation is similar to the catch bond-like behavior in other integrin types where the strength of the bond increases with the applied external force [148,149]. Using an AFM, the bond lifetime of a GPIbα-coated tip to a surface coating of vWF A1-domain was confirmed to have catch-bond behavior because the lifetime of the bond increased up to a peak load value and then reduced at higher loads as the catch-bond transitioned to a slip-bond [150]. Another significant study done using AFM was the direct measurement of retraction forces from a single platelet (Figure 5b) [27]. The AFM tip was integrated with a fluorescence microscope and the tip was coated with fibrinogen. This technique was not only able to measure the contractile force of single platelet, but it was also able to use AFM tips of different spring-constants to show that platelet regulate their retraction forces in proportion to the stiffness of the surround clot structure. These findings indicate that clot stiffening happens through the contraction of platelets as well as by the strain-stiffening of fibrin strands under the tension generated by platelet forces.

## 4. Conclusion and Future Directions

Platelet adhesion, aggregation, and clot retraction are important steps in hemostasis. Platelets are central to these steps and act as both an adhesive elements and as contractile units. Adhesion and aggregation happen at very small length-scales and therefore micro/nano devices have a promising capability to control and study the physical and chemical interactions for platelets. In particular, microfluidic devices have been developed to examine platelet function and clot formation under shear and micro/nano force probes have been used to study clot retraction. These devices allow for a degree of quantification and powerful insights at the molecular scale that was not possible with previous techniques. On the other hand, these devices are still under development and there are shortcomings associated with each of them. These devices are not simple to use or build. Moreover, few commercial sources are available for them so these techniques are not widespread in every lab, but those that are available are quite impressive [151–154]. As these technologies mature, it is likely that their use will become more widespread.

Micro/nano devices can provide new capabilities in studying platelets and hemostasis. They represent a programmable environment that can be modified to represent different *in vivo* conditions like shear, stiffness, or agonist concentration. Moreover, it may be possible to integrate the biomechanical assays that measure platelet forces with microfluidic devices in order to study the transitions from adhesion, aggregation, and clot retraction while also subjecting platelets to the biomechanical and biochemical triggers that occur *in vivo*. These types of assays can become useful for studies on genetic defects that affect the mechanobiology of platelets. Mutations in the GPIb-IX-V complex (Bernard-Soulier syndrome), integrin α_IIb_β_3_ (Glanzmann thrombasthenia), or the MYH9 gene that encodes the heavy chain of nonmuscle myosin IIA (May-Hegglin anomaly, Fechtner, Epstein, and Sebastian syndromes) can lead to serious bleeding disorders due to impaired adhesion, aggregation, or clot stability [155,156]. Biomechanical assays for platelets can help diagnose these clinical conditions and can open the door to new treatments for improved hemostasis.
